# Complete Genome Sequence of a *Burkholderia pseudomallei* Strain Isolated from a Pet Green Iguana in Prague, Czech Republic

**DOI:** 10.1128/genomeA.01761-16

**Published:** 2017-03-09

**Authors:** Mandy C. Elschner, Prasad Thomas, Hosny El-Adawy, Katja Mertens, Falk Melzer, Jan Hnizdo, Ivonne Stamm

**Affiliations:** a Friedrich-Loeffler-Institut, Federal Research Institute for Animal Health, Institute of Bacterial Infections and Zoonoses, Jena, Germany; b Animal Clinic, Bílá Hora, Prague, Czech Republic; c Vet Med Labor GmbH, Division of IDEXX Laboratories, Ludwigsburg, Germany

## Abstract

*Burkholderia pseudomallei* was isolated from pus from an abscess of a pet iguana living in a private household in Prague, Czech Republic. This paper presents the complete genome sequence of *B. pseudomallei* strain VB976100.

## GENOME ANNOUNCEMENT

*Burkholderia pseudomallei* causes melioidosis, a disease endemic in northern Australia and Southeast Asia. In Europe, cases of melioidosis were reported only from patients who had been traveling to regions endemic for the disease (1, 2). Pneumonia is the dominating clinical sign, followed by skin and soft tissue lesions, acute suppurative parotitis, and prostatitis in males (3). One predominant risk factor is diabetes mellitus type 2 (3). Besides humans, the bacterium has a very broad host range (4, 5). *B. pseudomallei* infections in pet iguanas were already reported in California, USA (6). In animals, acute and chronic forms of melioidosis are seen. Common symptoms in animals are anorexia, pyrexia, coughing, skin dehydration, and abscesses (5, 7). *B. pseudomallei* strain VB976100, isolated from pus of an abscess, was identified and characterized as described previously (8). For isolation of high-quality bacterial genomic DNA, the MasterPure Complete DNA and RNA purification kit (Epicentre, Illumina, Madison, WI) was used. Briefly, the bacterial cells were cultivated on nutrient agar containing 4% glycerol and harvested by rinsing with phosphate-buffered saline (PBS) (pH 7.2) solution. Nucleic acid extraction was performed as recommended by the manufacturer.

Genome sequencing was conducted by single-molecule real-time (SMRT) DNA sequencing (9) using the PacBio RSII sequencer generated from genomic DNA at GATC Biotech (Germany). Genome assembly was carried out using Hierarchical Genome Assembly Process (HGAP) algorithm version 3 (10) implemented in PacBio SMRT Portal version 2.3.0. The HGAP 3 assembly generated two contigs representing the genome for *B. pseudomallei* strain VB976100. The Gepard software (11) was used to determine the overlapping regions of circular sequences. Circlator (12) was used for the circularization of contigs. Finally, the circular contigs were polished with the RS_Resequencing.1 protocol in SMRT Portal version 2.3.0, and visualization was carried out using the SMRT View tool (PacBio).

The final circular chromosomes 1 and 2 were 4,090,973 bp and 3,190,235 bp, with G+C contents of 67.8% and 68.3%, respectively. The annotation (NCBI Prokaryotic Genome Annotation Pipeline) identified 6,148 protein-coding, 12 rRNA, three noncoding RNA (ncRNA), and 60 tRNA genes in the genome.

### Accession number(s).

This whole-genome sequencing project has been deposited at DDBJ/EMBL/GenBank under the accession numbers CP018054 (chromosome 1) and CP018055 (chromosome 2).
